# The Application of Genetic Risk Scores in Rheumatic Diseases: A Perspective

**DOI:** 10.3390/genes14122167

**Published:** 2023-12-01

**Authors:** Lotta M. Vaskimo, Georgy Gomon, Najib Naamane, Heather J. Cordell, Arthur Pratt, Rachel Knevel

**Affiliations:** 1 Department of Rheumatology, Leiden University Medical Center, 2333 ZA Leiden, The Netherlands; 2 Population Health Sciences Institute, Newcastle University, Newcastle upon Tyne NE2 4AX, UK; 3 Translational and Clinical Research Institute, Newcastle University, Newcastle upon Tyne NE2 4HH, UK; 4 Department of Rheumatology, Newcastle upon Tyne Hospitals NHS Foundation Trust, Newcastle upon Tyne NE7 7DN, UK

**Keywords:** genetic risk score (GRS), rheumatic diseases, genetics, clinical applicability, perspective

## Abstract

Modest effect sizes have limited the clinical applicability of genetic associations with rheumatic diseases. Genetic risk scores (GRSs) have emerged as a promising solution to translate genetics into useful tools. In this review, we provide an overview of the recent literature on GRSs in rheumatic diseases. We describe six categories for which GRSs are used: (a) disease (outcome) prediction, (b) genetic commonalities between diseases, (c) disease differentiation, (d) interplay between genetics and environmental factors, (e) heritability and transferability, and (f) detecting causal relationships between traits. In our review of the literature, we identified current lacunas and opportunities for future work. First, the shortage of non-European genetic data restricts the application of many GRSs to European populations. Next, many GRSs are tested in settings enriched for cases that limit the transferability to real life. If intended for clinical application, GRSs are ideally tested in the relevant setting. Finally, there is much to elucidate regarding the co-occurrence of clinical traits to identify shared causal paths and elucidate relationships between the diseases. GRSs are useful instruments for this. Overall, the ever-continuing research on GRSs gives a hopeful outlook into the future of GRSs and indicates significant progress in their potential applications.

## 1. Introduction

Genome-wide association studies (GWAS) have illuminated a diverse array of gene associations with rheumatic diseases [1]. They continue to promise insight into etiologic mechanisms, accelerated drug discovery, disease risk prediction, and precision medicine. However, modest effect sizes observed for individual associations have limited their clinical applicability. Genetic risk scores (GRSs) have emerged as a promising solution to this challenge. A GRS sums up the effects of all risk variants present in an individual and summarises their genetic risk profile for a given disease or other outcome of interest as a numerical value. The increased public availability of large-scale data repositories for accessing genetic information on well-phenotyped healthy and diseased people, exemplified by the UK Biobank, has led to considerable growth in GRS research.

**Figure 1:**
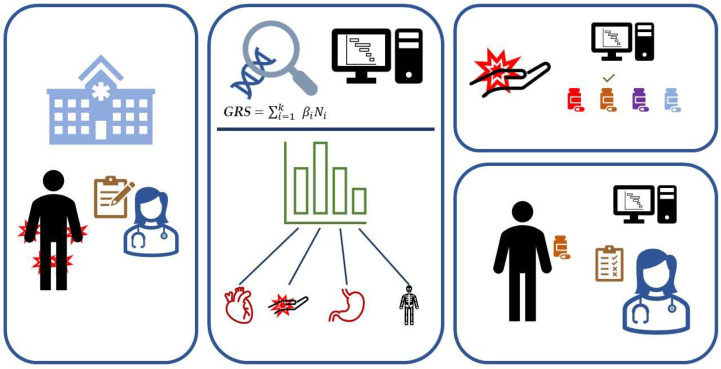
The application of genetic risk assessment in rheumatic diseases. When patients visit the outpatient clinic displaying symptoms like sore and swollen joints, it can be difficult for the clinician to diagnose the patient correctly. By utilizing genetic risk scores and electronic health records (EHR), it is possible to aid the clinician in determining the disease and thus be able to start the correct treatment, preventing permanent damage or disability.

Diseases like rheumatoid arthritis (RA) are complex and polygenic, meaning there are multiple genes, and thus multiple variants, affecting the probability of developing the condition. As a consequence, they are typically heterogeneous. Each variant can contribute more or less to overall disease susceptibility or progression. One trait can be associated with multiple variants, and one can contribute risk to more than one disease—potentially via a common mechanism. Numerous studies using GRSs have shown their potential value for predictive and/or discriminatory purposes by measuring the summed effect of multi-risk variants [2,3]. Since the symptoms of early RA are very similar to those of other rheumatic diseases, it is difficult for clinicians to correctly diagnose patients and deploy optimal management at the first visit, with consequent delays in disease control potentially contributing to permanent joint damage and/or disability. Genotype data are increasingly available, being patient-specific and time-independent; they have the potential to enhance clinicians’ ability to accurately diagnose this and other diseases (Figure 1), heralding better and more personalized care pathways [4]. In general, GRSs can be used not only for disease differentiation but also for investigating the relationship between two or more diseases, the interplay of GRS and the environment, the heritability and transferability of GRS between populations and disease stages, and detecting causal relationships.

In this perspective, we outline the principles behind GRSs and offer a comprehensive overview of their utilization in the rheumatic disease field. Our primary objective is to provide readers with a thorough understanding of the current GRS landscape in this setting; we furthermore seek to identify knowledge gaps in the existing literature that will inform future research.

## 2. How to Calculate a GRS

The term GRS is often used interchangeably with polygenic risk scores. Some differentiate the two, stating that PRSs incorporate the risk of many possibly correlated variants while GRSs contain only a smaller subset of proven associated variants. The difference between the two remains arbitrary, and the underlying principle is the same. Thus, we use, as others, GRS to refer to both.

A wide variety of tools have been developed to calculate GRSs, and Kachuri et al. review an extensive list of them [5]. The selection of the correct tool is dependent on the dataset and the nature of the research question that is to be answered. Each tool makes use of different algorithms to build the GRS. These can vary in terms of their tuning parameters and input data, resulting in completely different approaches for constructing the risk score.

The “classical” method for creating a GRS uses effect sizes, which are the calculated magnitudes of the associations with the trait [6], from GWAS summary statistics, prioritizing single nucleotide polymorphisms (SNPs) whose trait associations exceed a predetermined threshold of statistical significance [7]. At these loci, the sum of risk alleles (*k*) present in an individual is then calculated, each allele (*N*) being weighted according to its effect size (*β*) to derive the score (Equation (1)) [5,8]. Optimally, a validation step should be added to achieve the most reliable results. A very common way to validate the GRS is through internal validation, where the dataset is split using a resampling method (e.g., cross-validation), and the GRS is developed on each training set first and then validated in the corresponding test set by the calculation of the area under the receiver operating curve (AUC-ROC) [9,10]. A variation to internal validation is external validation, where the GRS is built on one cohort or dataset and then validated in another [11,12]. Calculating the GRS in this classical manner is beneficial since it is relatively fast and easy to interpret. In such cases, the SNP list should be adjusted to account for linkage disequilibrium (LD), such that multiple SNPs in LD become represented by a single variant [8,13]. SNPs are said to be in LD when their allelic concordance is higher or lower than expected under the assumption of statistical independence. In other words, when alleles of two different loci cooccur more often than one would expect by chance, these loci are said to be in LD with each other [14]. Various methods employed to account for LD between GRS SNPs to reduce signal bias have been described [15], each with pros and cons.

(1) $$\mathrm{GRS}=\sum_{i=1}^{k}\;\beta_{i}N_{i}$$

Among these, “LD pruning” removes SNPs in LD at random: their *p*-values and effect sizes are disregarded, meaning SNPs with the highest predictive utility may be inadvertently filtered out. “Clumping and thresholding” is a common alternative method in which SNPs most associated with the trait of interest are selected based on their low *p*-values. SNPs highly correlated with this initial selection will be removed, resulting in the inclusion of multiple independently operating SNPs that may be located in the same region [8]. A potential disadvantage, however, is that the thresholds used for clumping are often arbitrary.

Since trait-associated SNPs prioritized in GWASs do not necessarily represent those with the strongest association at a given locus, one can choose to apply shrinkage to the effect size estimates found in the GWAS. Shrinkage is a method where the effects of the SNPs are all moved towards the null in an attempt to obtain a more accurate estimation of the true size of the effect, increasing statistical power [16]. Shrinkage corrects for this uncertainty and leads to higher predictive accuracy in GRSs [8]. Shrinkage methods include Bayesian inference and penalized regression like LASSO and Ridge regression [13]. In LASSO regression, small effect sizes are reduced to zero, leaving SNPs that show a great absolute effect on the studied trait. In contrast, Ridge regression shrinks the largest effect sizes but does not shrink any effects to zero [8]. Which shrinkage method to select depends on the data at hand [8].

Non-classical approaches to obtaining a GRS include Bayesian methods. Although computationally more challenging, these methods have important advantages over classic methods, which, through the application of shrinkage, may discard information and thereby result in lower prediction accuracies [17]. Bayesian prediction methods can utilize genome-wide markers from GWAS summary statistics, rendering them more adaptive to different genetic architectures and, hence, more accurate [17]. For example, Ge et al.’s recently developed Polygenic Risk Score-Continuous Shrinkage (PRS-CS) approach uses GWAS summary statistics to build posterior effect sizes using an external LD reference panel. This is performed by adaptively shrinking the effect sizes using Bayesian regression (Equation (2)), where *Y* is the vector of traits, *x* is a matrix of genotypes, and *β* is the vector of effect sizes for these genotypes [17].

(2) $$Y_{N\;x\;1}=X_{N\;x\;M}{\;\beta }_{M\;x\;1}+\varepsilon_{N\;x\;1}$$

PRS-CS is, therefore, able to handle datasets of different genetic architectures and LD patterns [17]. This adaptive shrinkage causes smaller signals to be “shrunk” more since they are likely to be noise, whereas true signals for which a precedent exists in the literature will remain mostly untouched [17]. Simulation studies suggested this tool had advantages over other methods (including PRS-unadjusted, LD-pruning, *p*-value thresholding, LDrped-inf, and LDpred), particularly in large datasets.

Finally, Ma et al. classified SLE cases and controls using machine learning methods like artificial neural networks, random forests, and support vector machines. The prediction is often optimized by training on a high number of genetic variants. Ma et al. built the models using thousands of SNPs and compared their performance. The random forest model achieved an AUC-ROC of 0.84, which significantly performed better than the other models, and the GRS model reached an AUC-ROC of 0.74 [18]. These scores show great potential for applying machine learning in disease prediction.

## 3. What Is the GRS Used for, and Where Are We at for Rheumatic Diseases?

GRSs can be used to address a variety of clinically relevant questions, suggesting numerous potential applications. Table 1 displays an overview of the literature on the different applications that GRSs can be used for:

I. Prediction of a single disease of interest and/or disease outcomes;
II. Identification of genetic commonalities between two or more diseases;
III. Differentiation between two or more diseases;
IV. Exploring the interplay between GRSs and environmental factors;
V. Studying the heritability and transferability of GRSs in populations and disease stages;
VI. Detecting causal relationships using Mendelian randomization.

### 3.1. Prediction of Disease—Including Case Control Distinction, Treatment Effect, and Comorbidities

The most common way to utilize GRSs is to predict an individual’s susceptibility to a given trait or disease. Li et al. studied the capacity of GRSs to discriminate between cases of ankylosing spondylitis and healthy controls. They calculated the GRS using the MultiBLUP algorithm in LDAK [44] and validated its performance, demonstrating that it outperformed standard diagnostic tools, including sacroiliac MRI, CRP, or HLA-B27 testing [10]. Besides disease prediction, other aspects of disease can be studied as well. Reid et al. studied disease severity in systemic lupus erythematosus (SLE) cases and investigated the association with genetic risk. They compared groups with high and low GRS for SLE and found that a higher GRS is correlated with an earlier onset of disease, higher likelihood of organ damage, cardiovascular disease, and end-stage renal disease [39]. Comorbidities can be studied as well. Sandoval-plata et al. performed a case-control study on the transition of asymptomatic hyperuricemia (AH) to gout. Through GWAS, they identified 12 novel loci associated with the transition from AH to gout. A GRS model was generated from 17 SNPs in the discovery cohort and predicted in the replication cohort. The use of genetic and clinical predictors alone achieved accuracies (% cases correctly classified as having/not having gout) of 59% and 67%, respectively, which increased to 69% using a combined metric [23]. Adding genetics increases the accuracy of disease prediction compared to a clinician’s diagnosis. A GRS is a fairly simple formula that may fail to correctly predict under more complex architectures. Multi-omics approaches hold potential as a means of improving GRS prediction accuracy by capturing and incorporating additional biological information into predictive models [45]. A study by Shan et al. combined the GRS calculation tool LDpred with a transcriptional risk score (TRS), which showed improvements in AUCs after adding TRS [46]. Another way to achieve more complex models is by adding a machine learning approach to the GRS. Ma et al. tested several different machine learning approaches to improve GRS performance, of which the random forest model was able to improve the GRS model by 13%. The random forest AUC was 0.84, while the GRS AUC was 0.74 [18].

### 3.2. Identifying Genetic Commonalities between Diseases

An arguably more interesting hypothesis that can be studied using GRSs is to test the relation between two seemingly unrelated diseases or traits in an effort to uncover shared biological mechanisms. A GRS is calculated for the first disease, and, using statistical analyses like linear or logistic regression [22,26] or Mendelian randomization [25,31], the association of the GRS and the other disease GRSs and traits are compared and visualized. For example, the genetic risks of juvenile idiopathic arthritis and cardiovascular phenotypes were studied, and it was concluded that genetic susceptibility to juvenile idiopathic arthritis increases the risk for cardiovascular diseases [26]. Amongst older studies, Marquez et al. performed a large-scale meta-analysis on GWAS data for RA and found a new genetic risk linking rheumatoid arthritis with systemic lupus erythematosus. Hence, a variant at the component of the Oligomeric Golgi complex 6 (COG6) locus previously associated with RA and psoriasis has now been associated with SLE [47]. Zhang et al. made GRSs for two different groups of RA, namely one containing human leukocyte antigen (HLA) risk alleles and one not containing HLA alleles, and performed a phenome-wide association study (PheWAS) to study the association between 1317 traits from the UK Biobank (UKBB) and the RA GRSs. This resulted in significant associations with 13 immune-related traits, some of which were found with non-HLA GRS [37]. Maurits et al. used RA GRSs to test whether the factors that contribute to RA susceptibility also drive the transition from clinically suspected arthralgia patients (CSA) to arthritis [34].

### 3.3. Differentiation between Two or More Diseases

In our opinion, the most promising method for the clinical applicability of GRSs is to utilize these risk scores to differentiate between different diseases or disease subtypes. As mentioned, a core problem in diagnosing rheumatic diseases is that they are all based on symptoms experienced by the patient, which, in the case of rheumatic diseases, are very similar to each other. In relation to disease risk differentiation, Knevel et al. developed the G-PROB tool that transforms the GRSs of multiple rheumatic diseases into probabilities to facilitate the differentiation between these diseases. The G-PROB calculates the genetic risk scores considering the real-life disease prevalence as prior, which is then used to calculate the probability of having a disease given a certain symptom, assuming each individual has one of the diseases. Hence, amongst patients first presenting with unexplained inflammatory arthritis, accurate diagnoses could be discriminated from alternatives with a pooled AUC-ROC of >0.8 in some cases, apparently adding value to clinical evaluations [4]. Independent/prospective validation of tools such as this is awaited.

### 3.4. Interplay between GRS and Environmental Factors

Besides genetic effects, numerous other factors contribute to disease risk. Indeed, environmental and genetic determinants of complex disease heritability are only partially accounted for by identified risk factors, and a better understanding of interactions between genetic and environmental risk factors—which may be epigenetically mediated—could transform the field. GRSs can assist with exploring such gene–environment interactions. Current genetic diversity is caused by the interplay between genetics and the environment, where selection decides the survival of certain genotypes in populations, like, for example, the area in the genome where the largest genetic diversity is seen, the Major Histocompatibility Region (MHC) where the genes for B- and T-Cells are encoded in humans [48]. The study of this interplay between genetics and the environment (including factors such as air pollution, lifestyle choices, and socioeconomic status) could help us better understand mechanisms of genetic risk and, thus, ultimately help us understand complex disease-causation, onset, and course [48]. Thus, by combining and studying the genetic—and environmental risks, a greater understanding of complex diseases can be formed.

A study by Wells et al. found an association between an RA GRS and the presence of a particular gut microbiota implicated in RA pathogenesis, *Prevotella* spp. (a common organism found in the gut microbiome), in a population of individuals without the disease, suggesting that genetic influence on the microbiome may predate RA development. They concluded that healthy individuals displaying increased RA genetic risk might be predisposed to the presence of *Prevotella* spp. in their gut microbiota, with potential implications for understanding pathogenesis [12]. A study by Zhang et al. studied the impact of air pollutants on the risk of developing RA in >340,000 genotyped UK Biobank participants; here, a Cox proportional hazards model suggested exposure to multiple ambient air pollutants might increase disease risk more markedly in genetically susceptible individuals, although any gene-environment interaction was not statistically significant in this study [3]. The study of environmental factors remains challenging, however, not least because the dose (and hence impact) of environmental exposure can change over time.

### 3.5. Studying the Heritability and Transferability of Grss in Populations and Disease Stages

The power—and hence predictive utility—of a GRS is related to the heritability of the trait in question—the proportion of risk accounted for by genetic variability [43]. Assuming a 100% heritability would give the GRS maximal predictive power [13], but this is never achieved in common diseases. Mars et al., therefore, studied the extent to which a family history of non-communicable diseases overlapped with corresponding GRSs for the purpose of risk prediction, finding that they are complementary to one another rather than being interchangeable. Furthermore, it has been predicted that distinct heritage or ancestry will result in different GRSs due to differences in genetic architecture and LD patterns and that GRSs should, therefore, not be readily transferable between populations [43]. Privé et al. confirmed this by comparing GRSs between different global ancestries [7]. With the largest section of available genetic material currently being of European descent, the limited ethnicity problem is one of the biggest current drawbacks to the utilization of GRSs in clinical practice.

### 3.6. Detecting Causal Relationships Using Mendelian Randomisation

When answering causal questions based on observational data, a key untestable assumption is conditional exchangeability: all variables needed to adjust for confounding and selection bias must be identified and measured. Especially in observational data, this assumption is frequently not met. Instrumental variables (IVs) can be used to compensate for not knowing all confounders. IVs are variables that can allow us to identify the average causal effect of a certain variable, “A”, on a separate variable, “B”, even though not all confounders have been measured. IVs do this by being only associated with A and not (directly) with B, in this way isolating the causal effect of A on B. When using IVs, techniques exist to subsequently estimate the causal effect, including two-stage least squares estimation [49]. GRSs of medical conditions are good candidates for IVs in estimating causal relationships. Often, they follow the assumptions needed to function as IVs. They are (a) exclusively associated with one of the diseases, (b) do not directly affect the other disease, and (c) there is no shared cause between the GRS score of one disease and the other disease. The use of genetic factors as IVs to estimate causal effect is part of a framework known as Mendelian randomization. A study by Hindy et al. used the GRSs of osteoarthritis and several cardiometabolic risk factors to show that a causal relationship exists between these conditions [31]. Similarly, Lai et al., using Mendelian randomization, found that gout does have a causal effect on hypertension, but hypertension does not have a causal effect on gout [24]. Lastly, McCormick et al. found using GRS scores that hyperinsulinemia has a causal effect on hyperuricemia but not vice versa [25].

## 4. Discussion

### 4.1. Limitations and How to Overcome Them

Table 1 contains a list of the recent literature on GRS-related research in rheumatic diseases. We aimed for a comprehensive overview of the current literature, but not necessarily a complete overview. GRS research has seen many different applications in rheumatic diseases, yet some difficulties still affect all applications and diseases. A prominent one is the lack of availability of non-European genomic data. Different populations have different variants and LD patterns, making the application of GRSs to non-European ethnicities challenging. Currently, there are multiple attempts to diversify the availability of genetic material through the growth of biobanks and consortia, such as the National Institutes of Health (NIH)-funded Polygenic Risk Methods in Diverse Populations (PRIMED) Consortium, which pools genomic and phenotypic information from diverse populations [5]. When more diverse genetic data become available, the use of multi-ancestry data to perform GWASs could improve the transferability and accuracy of GRSs more generally. Ishigaki et al. utilized multi-ancestry GWAS outputs to calculate a GRS that achieved higher performance on multi-ancestry cases than a GRS calculated using a single-ancestry GWAS. Additionally, the multi-ancestry GRS achieved similar performance in European and East Asian populations as the corresponding single-ancestry GRS [15].

When assessing the GRS literature, particularly on disease prediction, it is important to realize that a high AUC-ROC does not mean a tool is useful in the clinic. It does not inform about the calibration of the probability, and if the calibration is suboptimal, a cutoff should be chosen. Finally, the AUC-ROC describes the group-level performance, while the individual-level performance (PPV and NPV) in the clinic is equally relevant. The AUC-ROC curves can show a good performance, while the test performs poorly due to imbalanced data (=low case prevalence). When the distribution of cases and controls within a study does not accurately reflect the situation in the population of its intended use, it can lead to a greatly altered prior probability of the disease of interest and impact the predictive accuracy of the model. Therefore, it is important to validate each tool within the population it is meant for. In the case of the G-PROB [4], the predictive accuracy was tested specifically within the population of patients with unexplained inflammatory arthritis, which aligns with the intended study population of the model. This validation approach targeted at the intended study population enhances the tool’s value for clinical applications and ensures that the predictive accuracy aligns with its use in the clinic.

Consideration of additional data sources in analyses, such as Electronic Health Record (EHR) data, lifestyle, and environmental factors, can help refine the accuracy of GRSs. In addition, knowledge of the variance explained by non-genetic factors is important- as this influences the predictive power of genetics, e.g., in a world where all environmental factors are identical for each individual, the remaining disease variance can be fully explained by genetics. Moreover, GRSs comprise mostly GWAS-identified common SNPs. Thus gene–gene interactions and rarer variants are not taken into account [13], which makes it difficult to predict the true heritability of diseases (and thus the maximal predictive power of a GRS).

### 4.2. Enabling Future Projects

Restrictions in the sharing of data make progress on GRS research logistically complicated. To obtain the input data for the GRS and to calculate the genetic risk for a singular individual, one needs individual-level genetic data. Privacy laws that safeguard the rights of research participants nonetheless place burdens on researchers, and the necessary sharing of such data to progress the field across large consortia, often involving multiple jurisdictions, compounds this. Other factors, including the challenge of accumulating genetic datasets from sufficiently large rare disease populations, also hinder the full utilization of GRSs [8].

### 4.3. Opportunities for Future Research

As can be seen from Table 1, a wide variety of tools and algorithms have been used to answer varying questions in rheumatic disease research. In this paper, we illustrated that the identification of disease and genetic commonalities between diseases are the most studied areas across rheumatic diseases. There is a relative dearth of research focussed on disease discrimination in relevant clinical contexts (such as the diagnostic setting), and we consider this an area of unmet need. For example, in the setting of the early arthritis clinic, clinical discrimination of osteoarthritis from immune-mediated inflammatory arthritis with a relatively low inflammation can be challenging. A validated, objective molecular discriminator could add valuable certainty to clinical evaluation and improve patient care. Applying GRS methods to leverage insight into gene–environmental interactions has also been under-studied. Moreover, though many tools have been developed, the search for and development of new methods to improve the predictive accuracy of GRSs continues.

GRS research further has the potential to better elaborate mechanisms of genetic risk for rheumatic diseases; this could, in turn, inform the design of preventative interventions for people at risk of these conditions in the future and/or mitigate against the development of multimorbidity. For example, Maurits et al. studied the difference in GRSs between RA cases, CSA cases, and healthy controls. Using the RA-susceptibility SNPs, they found a higher genetic RA risk for CSA cases who developed arthritis [34]. The difference was significant, but it was not high enough to obtain a good predictive ability. Possibly, other genes are involved in the transition from CSA to RA. A GWAS correcting for the known RA-GRS might unveil novel genes that play a role in this crucial stage of disease development.

In addition to a GRS, transcriptional risk scores (TRS) hold further potential to aid in unraveling mechanisms and pathogenesis of rheumatic diseases [45]. TRSs are a combination of risk alleles derived from GWASs and associated expression quantitative trait locus (eQTL) effects that, considered together, may contribute enhanced predictive accuracy over GRSs alone [50]. Combining different data levels for a multi-omics study shows potential to improve disease- and treatment-response prediction and could thus be used as stepping stones toward more personalized medicine [45]. A study by Shan et al. has already taken steps to this end, building a novel prediction framework by combining GRS and TRS, where TRS is defined as the weighted sum of imputed gene expression [46]. The TRSs, which were split into the single tissue TRS (STRS) and multi-tissue TRS (MTRS), achieved higher prediction accuracies in their respective categories than just the GRS alone, providing more evidence of the relevance of the multi-omics approach [45,46].

Since GRSs usually only capture simple genetic architectures, there is a recent rise in the application of machine learning algorithms in genetic risk score construction. A study by Ma et al. aimed to use machine learning to improve upon GRS models built using Lassosum, which has been proven to be one of the best tools for building GRS models [18]. The GRS model was then extended with three machine learning algorithms, namely random forest (RF), support vector machine (SVM), and artificial neural networks (ANN). The performances of the predictors were then validated using AUC. The RF model reached an AUC of 0.84, significantly outperforming the other predictors, with AUCs of 0.77 and 0.76 for SVM and ANN, respectively. However, all machine learning GRS models outperformed the GRS model, which reached an AUC of 0.74 [18]. These results show great potential for future studies to incorporate the RF GRS model to improve the predictions of complex diseases.

Thus far, most GRSs relied on known risk variants and built regression models using previously determined weights. A few studies show that a machine learning approach could improve risk prediction by either (or both) making optimal SNP selection and optimizing the risk weights. This fairly novel approach is still underexplored.

The co-occurrence of autoimmune diseases and comorbidities related to rheumatic diseases remains an incompletely explored field of medicine. For example, cardiovascular risk and rheumatic diseases. The current paradigm is that inflammation causes an increased risk of cardiovascular problems. By utilizing GRSs, Clarke et al. found that individuals with a high genetic risk for juvenile idiopathic arthritis are more likely to develop high-risk cardiovascular phenotypes [26]. This suggests that the two traits share a causal pathway instead of a relationship caused by having one of the diseases. Considerable effort is now being applied to expanding this research beyond isolated rheumatic diseases to IMIDs more generally and beyond. Such work would, where possible, increasingly adopt a multi-ancestry approach to improve both the accuracy of derived tools and equity amongst the populations it is designed to serve. As demonstrated by Ishigaki et al., such approaches also have the potential to further advance understanding of IMID biology [15].

### 4.4. Integrating GRSs into Healthcare Pathways

In an age of financially constrained public healthcare systems, the aspiration to deliver “precision medicine”—whereby customized patient care incorporates targeted disease prevention, streamlined referral pathways, and disease management recommendations that draw on personalized biomedical assessments—has gained traction as a means of optimizing societal wellbeing whilst minimizing cost. Coinciding with growth in eMedicine and rapid advances in data science capability, the extent to which EHR and bio-molecular data might synergize to improve medical decision making at each stage in the natural history of aging and multiple long-term conditions to improve care pathways is only now beginning to be explored. Against this backdrop, understanding the role of tailored GRS applications in pertinent clinical settings is in its infancy.

Since the transfer of GRSs between populations or ethnicities leads to low predictive accuracy, it is currently not possible to apply them fully in clinical care [5]. Variation in the accuracy of GRSs according to different ancestries (mentioned above) remains an important barrier to overcome since this would lead to inequity in the benefits of their clinical application as things stand [51]. While most GRSs are used for the prediction of disease risk, there has been little focus on the link between treatment response and genetics. GRSs in breast cancer and cardiovascular diseases have demonstrated the potential for their application in this way [51]. This subject remains under-studied in rheumatic diseases, with genetic associations with treatment efficacy displaying only weak effects to date [52]. Combining different omics together in a larger study might improve power in regards to the prediction of treatment response [45]. In other fields, the GRSs have already demonstrated potential clinical applicability. Thanos et al. performed a longitudinal study combining psychosocial questionnaires and genetics from a population of bariatric surgery patients to predict negative outcomes. They were able to construct GRSs for individual patients, which could warn them about the increased risk of poor outcomes related to addictive behavior risk [53]. A follow-up study by Thanos et al. found that carrying certain alleles and psychological traits would correlate with greater weight loss after bariatric surgery [54]. By combining GRSs with other tools, these studies highlight promising developments in the realm of precision medicine.

With this in mind, the SPIDeRR consortium (Stratification of Patients using advanced Integrative modeling of Data Routinely acquired for diagnosing Rheumatic complaints) aims to leverage EHR data from seven countries to improve the “journey” of people presenting with musculoskeletal complaints suspected to have a rheumatic disease. The project aspires to apply GRS tools with the potential to discriminate diseases and disease subsets (such as G-PROB [4]) to appraise their utility in healthcare settings, both using traditional GRS tools and optimizing prediction through machine learning. SPIDeRR will furthermore explore the extent to which such tools might add value to those developed from EHR data by applying artificial intelligence for diagnosing and prognosticating people with musculoskeletal symptoms.

Besides disease differentiation, SPIDeRR will also investigate the interplay between genetics and the environment. For example, longitudinal follow-up of people already presenting with unfavorable genetic risk profiles might reveal important environmental determinants of morbidity according to whether adverse outcomes ensue. This could give more insight into possible causal environmental risk factors on disease susceptibility, trajectory, and recovery. In addition to conferring disease risk, genetic variants can be protective. By studying protective variants, one can identify genetic pathways that are useful or necessary for remission. This could further strides toward more personalized management.

### 4.5. Concluding Remarks

Though genetic studies face many challenges regarding data availability, transferability, and applicability, many efforts are being made to improve science and clinical care using GRSs. The collection of genetic data, including the variety of people and diagnoses as seen in the clinical real world, would tackle the greatest problem when it comes to genetics research thus far. We believe genetics through GRSs can further optimize the identification of diseases and etiologic mechanisms. Genetic risk scores offer clinicians a quick and easy individual-level overview of an individual’s genetic risk for specific diseases. This makes the interpretation of GWAS results easier, allowing clinicians to utilize these strong evidence-based results for patient benefit, making strides towards improving medicine at the patient level.

## Figures and Tables

**Table 1:** An overview of the literature on the different applications of GRSs. This list contains papers from 2019 and later following a non-exhaustive literature research. The category numbers behind the stated diseases correspond with the category of applications mentioned earlier.

Paper	Disease	Population	Results
Ko et al., 2022 [19]	AS (I)	A Taiwanese cohort was used for both training and testing purposes.	GRS was used to predict AS versus healthy controls. Of all GRS methods used, GenEpi had the highest performance with an AUC-ROC of 0.81, while LDpred2 had the lowest performance with an AUC-ROC of 0.76.
Li et al., 2021 [10]	AS (I)	Data were used comprising individuals of European, Chinese, Turkish, and Iranian descent.	GRSs performed better in disease prediction than other standard diagnostic tests for predicting AS versus healthy controls, with an AUC-ROC of 0.92.
Chang et al., 2022 [20]	Gout/AH (I)	A Taiwanese cohort was used, classifying individuals as either gout, AH, or normouricaemic.	A positive correlation was found between gout/AH GRS and the development of gout/AH as compared to normouricemia.
Lin et al., 2023 [21]	Gout/AH (I)	A Taiwanese cohort was used, classifying individuals as either gout, AH, or normouricaemic.	GRS explained 0.59–0.89% of the variance of the phenotype of gout/AH in females.
Sumpter et al., 2023 [22]	Gout (II)	Seven European, two East Polynesian, and one West Polynesian gout cohorts were used.	Gout GRS was associated with earlier onset of gout and tophaceous disease in men but not in women.
Sandoval-Plata et al., 2021 [23]	Gout vs. AH vs. healthy control (I, III)	The UK biobank was used, classifying individuals as either gout, AH, or normouricaemic.	Predicting gout versus AH in a hyperuricemic population using GRS gave an AUC-ROC of 58.5% while adding GRS to a model with demographic risk factors increased the AUC from 66.7% to 69.2%.
Lai et al., 2022 [24]	Gout and hypertension (VI)	The Taiwanese biobank was used.	Gout susceptibility seems to have a causal effect on hypertension. Hypertension, however, does not have a causal effect on gout.
McCormick et al., 2021 [25]	Hyperuricemia and hyperinsulinemia (VI)	Multiple available GWAS cohorts were used, including the CKDGen, MAGIC, and UKBiobank cohorts.	Provide evidence that hyperinsulinemia has a causal effect on hyperuricemia but not vice versa.
Cánovas 2020 [11]	JIA (I)	Three JIA cohorts from the UK, US, and Australia were used. Only the UK cohort was employed as a training set.	A model predicting JIA versus healthy controls based on JIA GRS achieved AUC-ROC’s of 0.67, 0.66, and 0.67 for the UK, US, and Australia cohorts, respectively. The highest prediction was seen for the oligoarthritis JIA subtype.
Clarke et al., 2022 [26]	JIA and Cardiovascular risk factors (II)	The ALSPAC cohort, a UK cohort encompassing mainly individuals of European descent, was used.	The GRS for JIA is associated with multiple cardiovascular risk factors, including higher diastolic blood pressure, blood insulin levels, insulin resistance index, log hsCRP, waist circumference, fat mass index, and body mass index.
Lacaze et al., 2022 [27]	OA (I)	An Australian cohort was used with patients of European descent.	Higher GRS was associated with an increased knee and hip replacement risk. Adding GRS to a prediction model for knee replacement (that includes multiple other variables) increased the AUC-ROC from 0.666 to 0.668. An AUC increase from 0.57 to 0.59 was observed for a similar model for hip replacement.
Morita et al., 2023 [28]	OA (I)	Both a Japanese cohort and a multi-ancestry cohort were used to construct the GRS. The GRS score was evaluated on the Japanese population.	The addition of a GRS based on multi-population GWAS to a prediction model with traditional risk factors increased the AUC-ROC from 0.74 to 0.75 (*p* = 0.03) on the prediction of OA versus controls.
Sedaghati-Khayat et al., 2022 [29]	OA (I)	Three Dutch cohorts were used, with OA cases and healthy controls.	Predicting OA using GRS yielded an AUC-ROC of 0.57 while adding GRS to a prediction model with clinical risk factors increased the AUC from 0.64 to 0.66. GRS was found to be associated with radiographic OA, clinical OA, and radiographic OA progression. A stronger association was found with clinical OA as compared to radiographic OA.
Zhu et al., 2022 [30]	OA (IV)	The UK biobank was used.	Various types of physical activity are associated with hip/knee OA. There is no interaction between types of physical activity and OA GRS on hip/knee OA, meaning that the effect of physical activity on OA was the same across individuals with different genetic susceptibilities.
Hindy et al., 2019 [31]	OA and cardiometabolic risk factors (VI)	The Malmö Diet and Cancer Study cohort and the UK Biobank were used.	Using Mendelian randomization, evidence is provided that elevations in low-density lipoprotein cholesterol reduce the risk of OA while also showing that an increased BMI increases the risk of OA.
Smith et al., 2020 [32]	PsA (III)	A US cohort containing individuals with either psoriasis only or psoriasis with PsA was used.	Weighted GRS achieved an AUC-ROC of 56.2% in discriminating between psoriasis with and without PsA in a population with psoriasis, with an AUC of 56.9% in an HLA-only model.
Honda et al., 2022 [33]	RA (I)	A large Japanese cohort was used for both training and testing.	The GRS based on RA susceptibility SNPs is significantly associated with the severity of radiographic progression within RA, with AUC-ROC values of 0.56–0.66.
Ishigaki et al., 2022 [15]	RA (I)	Multi-ancestry data was used from 37 cohorts, including individuals from European, East Asian, African, South Asian and Arab ancestries.	GRS based on multi-ancestry GWAS outperformed single-ancestry GWAS-based GRS in predicting multi-ancestry RA cases versus healthy controls. The multi-ancestry GRS had an AUC-ROC of 0.59–0.66.
Maurits et al., 2023 [34]	RA, CSA (I, II)	Individuals of European descent were included and classified as either RA, CSA, or healthy controls.	RA GRS differed significantly between healthy controls, CSA, and RA patients, increasing across these groups. Similar results were observed for the HLA-shared epitope.
Jones et al., 2019 [35]	RA and Cognitive/Psychiatric phenotypes (II)	The ALSPAC cohort, a UK cohort encompassing mainly individuals of European descent, was used.	There is an association between the GRS of RA and a lower total IQ at age 8, as well as symptoms of hyperactivity and inattention at ages 4–16.
Kasher et al., 2022 [36]	RA & Osteoporosis (II)	The UK biobank was used, selecting only individuals of European descent.	RA GRS is associated with osteoporotic fractures as well as total-, spine-, and forearm bone mineral density. It seems that pleiotropy is involved in the association between RA and osteoporosis.
Zhang et al., 2023 [37]	RA (II)	The UK biobank was used, only selecting individuals without evidence of RA.	Studied the association between RA GRS and multiple phenotypes. A total of 13 out of 20 phenotypic traits that were associated with the RA GRS were autoimmune-related traits.
Wells et al., 2020 [12]	RA (IV)	Cohorts from the UK and Switzerland were used, and individuals with RA were excluded from the analysis.	GRS for RA is positively associated with the gut bacteria *Prevotella* spp. This bacteria is also associated with preclinical RA phases in non-RA individuals.
Zhang et al., 2023 [3]	RA (IV)	The UK biobank was used, including only individuals of European descent.	Air pollution was positively associated with the risk of developing RA. The interaction between air pollution and the RA GRS score was not significant, although the risk of developing RA in individuals with the highest genetic risk score and highest air pollution exposure was twice that compared to individuals in the lowest-risk categories.
Kwon et al., 2023 [38]	SLE (I)	A Korean SLE cohort, encompassing patients with different SLE manifestations, was used.	A high GRS was associated with an earlier age of onset, higher anti-Sm antibody levels, and the development of LN. Predicting LN using GRS had an AUC-ROC of 0.56–0.60.
Ma et al., 2022 [18]	SLE (I)	Individuals of Chinese and European ancestry were studied, with SLE cases and healthy controls.	GRS achieved an AUC-ROC of 0.74 in discriminating SLE vs. healthy controls. A random forest method was developed that improved the AUC to 0.84. Machine learning shows potential for genetic prediction.
Chen et al., 2020 [9]	SLE (I)	Three European and two Chinese cohorts with SLE cases and healthy controls were studied, both for training and testing purposes.	GRS predicts SLE (versus healthy controls) and differentiates between SLE with and without renal disease. The AUC-ROC are 0.64–0.72 and 0.58–0.62, respectively.
Reid et al., 2019 [39]	SLE (I)	A Swedish cohort was used with SLE cases and healthy controls.	The AUC-ROC of predicting SLE using GRS was 0.78. A high GRS is associated with worse survival, organ damage, cardiovascular disease, proliferative nephritis, ESRD, and antiphospholipid antibodies in patients with SLE.
Tangtanatakul et al., 2020 [40]	SLE (I)	Data based on a Chinese cohort was used to construct the GRS, which was subsequently tested on a Thai cohort.	GRS constructed using data from a Chinese cohort yielded an AUC-ROC of 0.76 in discriminating SLE versus healthy controls when applied to a cohort from the Thai population.
Wang et al., 2021 [41]	SLE (I)	Data based on a European cohort was used to construct the GRS, which was subsequently tested on a Chinese cohort.	GRS was used to predict SLE versus healthy controls. Lassosum achieved an AUC-ROC between 0.62 and 0.64. LDpred produced similar results. Ancestry-matched predictors perform better with an AUC of 0.76.
Kawai et al., 2021 [42]	SLE & cardiometabolic disorders (II)	A US cohort was used, restricting the analysis to individuals of European descent.	SLE GRS was associated with an increased risk of type 1 diabetes and several other autoimmune phenotypes but not cardiometabolic disorders
Knevel et al., 2020 [4]	RA, SLE, SpA, PsA and gout (III)	Three US databases of ICD codes were used, only selecting individuals who were self-reported as white.	A new tool was developed to distinguish between multiple related rheumatic diagnoses (RA, SLE, SpA, PsA, and gout). This tool was tested on three independent cohorts, achieving AUC-ROCs of 0.69, 0.81, and 0.84. For all patients, at least one diagnosis could be ruled out, and in 45% of patients, a likely diagnosis was identified with a PPV of >64%.
Mars et al., 2022 [43]	Multiple diseases (V)	FinnGen study data, a large Finnish network combining genomic information with national health registries, was used.	Family history (FH) and GRS are independent and not interchangeable measures; instead, they provide complementary information on inherited disease susceptibility. GRS explains, on average, 10% of the effect of 1st-degree FH, and 1st-degree FH explains 3% of GRS. In most diseases, a high genetic risk combined with positive family history is associated with a higher risk. A low genetic risk compensated for risk through a positive family history.

Methodologic application categories: I = Prediction of a single disease of interest and/or disease outcomes; II = Identification of genetic commonalities between two or more diseases; III = Differentiation between two or more diseases; IV = Exploring interplay between GRSs and environmental factors; V = Studying the heritability and transferability of GRSs in populations and disease stages; VI = Detecting causal relationships using mendelian randomisation; AS—Ankylosing Spondylitis, GRS—Genetic Risk score, AUC-ROC—Area Under the Receiver Operating Characteristic Curve, AH—Asymptomatic Hyperuricemia, HLA = Human Leukocyte Antigen; hsCRP = high sensitivity C-reactive Protein; JIA = Juvenile Idiopathic Arthritis; LD = Linkage Disequilibrium; JIA—Juvenile Idiopathic Arthritis, UK—United Kingdom, US—United States, OA—Osteoarthritis, PsA—Psoriatic Arthritis, RA—Rheumatoid Arthritis, CSA—Central Serous Atrophy, IQ—Intelligence Quotient, SLE—Systemic Lupus Erythematosus, LN—Lupus Nephritis, ESRD—End-Stage Renal Disease, ICD codes—International Classification of Diseases codes, PPV—Positive Predictive Value, FH—Family History, FinnGen—Finnish Genetics Study, CSA = Clinically Suspected Arthralgia; CV = Cardiovascular Diseases; GWAS = Genome Wide Association Study; SNP = Single Nucleotide Polymorphism; SpA = Spondyloarthropathy; PsA = Psoriatic Arthritis.

## Data Availability

No new data were created or analyzed in this study. Data sharing is not applicable to this article.
